# Unveiling the role of transgelin as a prognostic and therapeutic target in kidney fibrosis via a proteomic approach

**DOI:** 10.1038/s12276-024-01319-7

**Published:** 2024-10-07

**Authors:** Soie Kwon, Seongmin Cheon, Kyu-Hong Kim, Areum Seo, Eunjin Bae, Jae Wook Lee, Ran-Hui Cha, Jin Ho Hwang, Yong Chul Kim, Dong Ki Kim, Yon Su Kim, Dohyun Han, Seung-Hee Yang

**Affiliations:** 1 Department of Internal Medicine, Chung-Ang University Hospital, Seoul, Republic of Korea; 2 Department of Internal Medicine, College of Medicine, Chung-Ang University, Seoul, Republic of Korea; 3 Department of Clinical Medical Sciences, Seoul National University, Seoul, Republic of Korea; 4 School of Biological Sciences and Technology, Chonnam National University, Gwangju, Republic of Korea; 5 Kidney Research Institute, Seoul National University College of Medicine, Seoul, Republic of Korea; 6 Department of Biomedical Sciences, College of Medicine, Seoul National University, Seoul, Republic of Korea; 7 Department of Internal Medicine, Gyeongsang National University College of Medicine, Gyeongsang University Changwon Hospital, Gyeongsang, Republic of Korea; 8 Nephrology Clinic, National Cancer Center of Korea, Seoul, Republic of Korea; 9 Biomedical Research Institute, Seoul National University Hospital, Seoul, Republic of Korea; 10 Department of Internal Medicine, Seoul National University Hospital, Seoul, Republic of Korea; 11 Department of Internal Medicine, Seoul National University, College of Medicine, Seoul, Republic of Korea; 12 Proteomics Core Facility, Biomedical Research Institute, Seoul National University Hospital, Seoul, Republic of Korea; 13 Department of Transdisciplinary Medicine, Seoul National University Hospital, Seoul, Republic of Korea; 14 Department of Medicine, Seoul National University College of Medicine, Seoul, Republic of Korea

**Keywords:** Predictive markers, End-stage renal disease

## Abstract

Chronic kidney disease (CKD) progression involves tubulointerstitial fibrosis, a process characterized by excessive extracellular matrix accumulation. To identify potential biomarkers for kidney fibrosis, we performed mass spectrometry-based proteomic profiling of human kidney tubular epithelial cells and kidney tissue from a 5/6 nephrectomy rat model. Multidisciplinary analysis across kidney fibrosis models revealed 351 differentially expressed proteins associated with kidney fibrosis, and they were enriched in processes related to the extracellular matrix, kidney aging, and mitochondrial functions. Network analysis of the selected proteins revealed five crucial proteins, of which transgelin emerged as a candidate protein that interacts with known fibrosis-related proteins. Concordantly, the gene expression of transgelin in the kidney tissue from the 5/6 nephrectomy model was elevated. Transgelin expression in kidney tissue gradually increased from intermediate to advanced fibrosis stages in 5/6 Nx rats and mice with unilateral ureteral obstruction. Subsequent validation in kidney tissue and urine samples from patients with CKD confirmed the upregulation of transgelin, particularly under advanced disease stages. Moreover, we investigated whether blocking TAGLN ameliorated kidney fibrosis and reduced reactive oxygen species levels in cellular models. In conclusion, our proteomic approach identified TAGLN as a potential noninvasive biomarker and therapeutic target for CKD-associated kidney fibrosis, suggesting its role in modulating mitochondrial dysfunction and oxidative stress responses.

## Transgelin: a promising biomarker for chronic kidney disease fibrosis

Chronic kidney disease is caused by kidney fibrosis, where healthy kidney tissue becomes scar, affecting kidney function. This research aimed to find noninvasive signs of kidney fibrosis by studying proteins in human kidney cells and animal CKD models. They used mass spectrometry, a method to identify and quantify proteins, to find potential signs in the body fluids that could show kidney fibrosis without needing kidney biopsy. The study found that the protein TAGLN increases in kidney tissue and urine in CKD conditions, suggesting it could be a useful sign of kidney fibrosis. Further tests showed that blocking TAGLN could reduce kidney fibrosis, indicating its potential as a target for new treatments. The researchers conclude that TAGLN is a promising sign of kidney fibrosis and could lead to better diagnostic and treatment options for CKD patients.

This summary was initially drafted using artificial intelligence, then revised and fact-checked by the author.

## Introduction

Chronic kidney disease (CKD) progression shares a final common pathway of kidney fibrosis, regardless of the underlying cause^1^. Fibrosis involves the accumulation of excessive tubulointerstitial extracellular matrix (ECM) and is considered an aging process^2–4^. Partial epithelial-to-mesenchymal transition involves the acquisition of mesenchymal properties in tubular epithelial cells, and it is a pivotal process in kidney fibrosis^5^. An increase in mitochondrial structural and functional impairments contributes to CKD progression^6^. These impairments further promote tubulointerstitial fibrosis, emphasizing the crucial role of mitochondrial involvement in CKD and the kidney aging process^4,7^.

Noninvasive kidney function estimation depends on measuring serum creatinine and cystatin C levels and evaluating proteinuria. However, these parameters have limitations in accurately reflecting the extent of kidney fibrosis in certain clinical situations and diseases^8–10^. Consequently, efforts have been made to identify novel biomarkers commonly associated with kidney fibrosis. Recent studies have used various high-throughput approaches, including bulk and single-cell RNA sequencing, to identify promising biomarkers^8,11^. Despite efforts to validate these biomarkers in diverse cohorts, the lack of reproducibility and the heterogeneous results obtained from different samples have prevented their successful translation to preclinical and clinical applications^10^, necessitating further research to identify reliable diagnostic and therapeutic biomarkers.

Biomarker evaluations based on kidney tissue directly correlate with kidney fibrosis; invasive procedures limit its use in clinical practice. Therefore, interest in exploring noninvasive biomarkers derived from urine and blood samples is increasing^10,12^. However, the utilization of urine samples is hindered by variations in biomarker concentrations and challenges in identifying the specific origin of the detected substances. In contrast, blood samples can reflect changes in multiple organs, making biomarker evaluations more complex^3,13^. High-throughput gene expression profiling methods, including transcriptomic and proteomic approaches, have emerged as valuable tools for investigating specific diseases^14–16^. However, novel biomarkers of kidney fibrosis have received limited attention. We previously applied a proteomic approach to analyze multiple specimen types concurrently, including urine from patients with CKD, kidney tissue from a cellular model of hypoxia-induced kidney fibrosis, and a 5/6 Nx rat model, to address the limitations associated with each sample type and to yield comprehensive insights^17^.

Building upon the strengths of this multi-sample study, we used a reverse approach to enhance protein discovery and provide insights into renal origin. We conducted a mass spectrometry (MS)-based proteomic study to identify potential biomarkers of kidney fibrosis using primary cultured human-derived kidney cells and kidney tissue from CKD animal models, with subsequent evaluation using kidney and urine samples from patients with CKD.

## Materials and methods

### Preparation of human primary tubular epithelial cells (hTECs)

Normal kidney tissue was collected from patients who underwent nephrectomy for renal cell carcinoma and was carefully dissected to isolate the proximal renal tubular segments. The segments were minced and digested using Hank’s balanced salt solution containing 1.5 mg/mL collagenase (Sigma‒Aldrich, St. Louis, MO, USA) at 37 °C for 1 h. The digested cells were washed through a series of sieves (150, 120, 70, and 40 μm) with 1× phosphate-buffered saline (PBS) and centrifuged at 500 × *g* for 5 min. The cells were recovered from the pellet and incubated in DMEM/F12 (Lonza, Basel, Switzerland), and the tubules floating in the media were collected and cultured on collagen-coated Petri dishes (BD Biosciences, Franklin Lakes, NJ, USA) until epithelial cell colonies had formed. Additionally, hTECs were sorted using a fluorescence-activated cell sorting Calibur instrument (BD Biosciences) with fluorescein isothiocyanate-labeled anti-AQP1 staining (Abcam, Cambridge, MA, USA). The sorting process was performed at 4 °C for 3 min. Subsequently, the hTECs were used after 2–3 passages^18^. The Institutional Review Board (IRB) of Seoul National University Hospital reviewed and approved the protocol for obtaining and processing human kidney samples (SNUH, IRB No. 2110-026-1260).

### Fibrocellular model: induction and blockade of hTECs

**Fig. 1:**
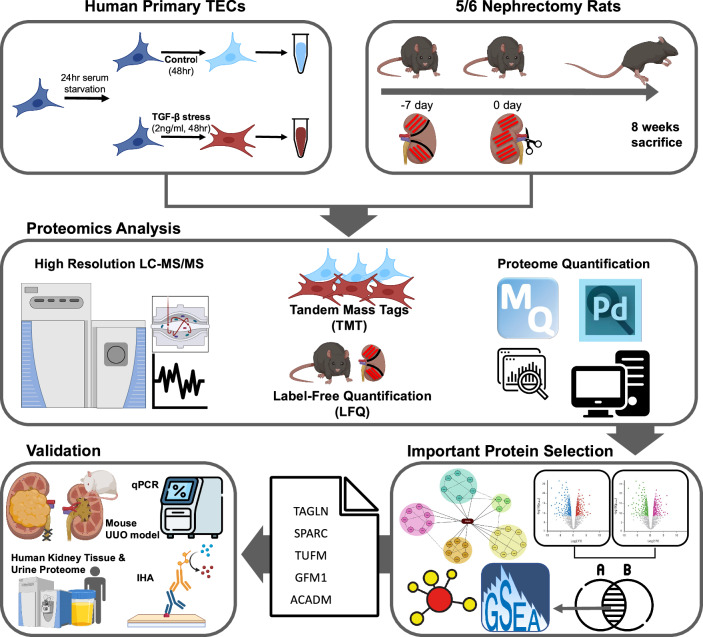
Schematic of MS-based proteomics in this study. Proteomic analysis of primary human tubular epithelial cells and rat kidney tissues. Differentially expressed proteins were identified to determine altered protein expression after fibrosis. The commonly regulated proteins were identified through cross-validation between the models and were validated using various molecular experiments. MS mass spectrometry.

Purified hTECs were cultured with 2 ng/mL recombinant transforming growth factor β (rTGF-β) for 48 h to generate a fibrous cellular model, and a control model was cultured without rTGF-β stress (Fig. 1). After 48 h, each group of hTECs was harvested with PBS and subjected to protein extraction. A TAGLN-blocking peptide (iTAGLN; LS-E8376, LS Bio, Shirley, MA, USA) was used to evaluate the therapeutic potential of TAGLN. iTAGLN was administered simultaneously with rTGF-β at concentrations of 0.25, 0.5, or 1.0 μg/mL for 48 h in the treatment group.

### Animal model: 5/6 nephrectomy (Nx) rat model

Eight-week-old male Sprague-Dawley rats purchased from Jackson Laboratory (Bar Harbor, ME, USA) were used in this study. The rats were randomly assigned to either the 5/6 nephrectomy (5/6 Nx) model group or the control group (n = 4 per group). Anesthesia was induced via a mixture of ketamine (100 mg/mL) and xylazine (25 mg/mL) before all surgical procedures. The 5/6 Nx was performed in a two-step manner. First, the left kidney was completely removed. Then, the lower third of the right kidney was resected through ligation of the supplying renal artery, and the upper third was resected through electrocoagulation. One week later, a total left nephrectomy was performed by ligation of the renal vascular pedicle, marking day 0 for the 5/6 Nx model. For the control animals, sham operations involving flank incisions to expose but not resect the kidneys were performed on the same operative dates, with durations similar to those of the 5/6 Nx procedures.

The rats were placed in metabolic cages, and their systolic blood pressure (SBP), body weight, and blood and urine samples were obtained 4 and 8 weeks after the index date. Their SBP was measured via tail-cuff method with a CODA system (Kent Scientific Co., Torrington, CT, USA). Serum creatinine and blood urea nitrogen levels were measured using a Hitachi 747 (Hitachi, Tokyo, Japan). The rats were euthanized 8 weeks after the left nephrectomy, and the kidneys were harvested^19^. The Institutional Animal Care and Use Committee (IACUC) of SNUH reviewed and approved the study protocol involving the 5/6 Nx rat model (IACUC No. 18-0222-S1A0).

### Unilateral ureteral obstruction mouse model

Seven-week-old male C57BL/6 mice were purchased from Koatech (Seoul, South Korea). After anesthesia, a left flank incision was made to expose the left ureter. Subsequently, the exposed left ureter was ligated with 5-0 silk to create an obstruction and prevent urinary flow. The control group underwent the same surgical procedures without ureter ligation. The mice were euthanized at 3, 7, and 14 days post-surgery^20^. The IACUC of SNUH reviewed and approved the study protocol involving the unilateral ureteral obstruction (UUO) mouse model (IACUC No. 21-0243-S1A0).

### Kidney biopsy and urine sample acquisition from patients with CKD

Ten formalin-fixed renal biopsy samples were obtained from patient groups categorized based on the CKD epidemiology collaboration-estimated glomerular filtration rate (eGFR) at the time of renal biopsy. The patients were classified into CKD stage 1/2 (eGFR ≥ 60 mL/min/1.72 m^2^), stage 3 (30 mL/min/1.72 m^2^ < eGFR ≤ 60 mL/min/1.72 m^2^), and stage 4/5 (eGFR ≤ 30 mL/min/1.72 m^2^). The baseline characteristics of the participants are summarized in Supplementary Table 1. In these patients, correlations between several conventional serum and urine laboratory variables and kidney tissue TAGLN expression were evaluated. We validated the TAGLN using urine proteomics results from the CKD patients in our previous study, which were different from the biopsy patients^17^.

The human urine and kidney biopsy samples used in this study were sourced from a prospective biobank, which had received prior approval from the IRB (IRB No. 1710-058-894). The IRB of SNUH reviewed and approved the study protocol involving human-derived samples (IRB No. 2201-136-1294).

### Sample preparation for MS analysis

The hTECs and rat kidney tissues were subjected to proteomic sample preparation for isobaric or label-free quantification. Total protein was extracted from primary cells and kidney tissues using a lysis buffer (4% sodium dodecyl sulfate [SDS], 2 mM tris(2-carboxyethyl) phosphine hydrochloride [TCEP], and 100 mM Tris-HCl; pH 7.5) with sonication. The protein extracts were heated at 95 °C for 30 min. The protein concentration was measured using a reducing agent-compatible BCA assay kit (Pierce, Waltham, MA, USA). Proteins derived from tissues or cells were digested using filter-aided sample preparation (FASP), as described previously^21,22^. Briefly, 150 μg of protein was precipitated using cold acetone, and the pellets were dissolved in 2% SDS-containing buffer (2% SDS, 10 mM TCEP, 40 mM chloroacetamide, and 0.1 mM Tris-HCl; pH 8.5). After boiling at 95 °C for 20 min in the dark, the resulting denatured and alkylated proteins were loaded onto a 30 kDa spin filter (Merck Millipore, Darmstadt, Germany). Then, UA buffer (8 M urea in 0.1 M Tris-HCl, pH 8.5) was added to this spin filter, which was subsequently centrifuged. The buffer was replaced with 300 µL of UA solution twice using centrifugation to remove the SDS. The UA buffer was subsequently exchanged with 50 mM HEPES three times by centrifugation. The proteins were digested overnight with trypsin/Lys-C (enzyme-to-protein ratio [w:w] 1:100) at 37 °C. Lastly, the digested peptides were collected in a new tube via centrifugation. Sequential elution was performed using 50 mM HEPES and 0.5 M NaCl. Peptide concentrations were measured by tryptophan fluorescence assay^23^. The peptides obtained from the hTEC samples were subjected to TMT labeling, and the peptides obtained from the 5/6 Nx rat model samples were acidified with 10% trifluoroacetic acid (TFA; Thermo Fisher Scientific, Waltham, MA, USA). A 3-fractionation strategy was employed to increase the proteome depth of the 5/6 Nx rat model samples. The acidified peptides were loaded onto homemade C18-sulfonated styrene-divinylbenzene polymer (C18-SDB-RPS) StageTips (3 M, St. Paul, MN, USA) following previously described procedures^24^. The fractionated peptides were completely dried in a vacuum dryer and stored at –80 °C until further analysis.

### TMT labeling

For the hTECs, TMT labeling was performed according to the manufacturer’s protocol with some modifications^25^. First, 0.8 mg of TMT reagent (Thermo Fisher Scientific) was dissolved in 100% acetonitrile (ACN), after which 25 μL of this TMT solution was added to 25 μg of peptide sample along with ACN, resulting in a final concentration of 30% (v:v). As an internal standard, 270 ng of peptide derived from ovalbumin was spiked into each sample. After incubation at room temperature for 90 min with shaking, the reaction was quenched by adding 5% hydroxylamine to achieve a final concentration of 0.3% (v:v). TMT-labeled samples were pooled in the same volume for all samples. The resulting peptide mixtures were vacuum-dried in a centrifuge and desalted using an HLB OASIS column (Waters, Milford, MA, USA) according to the manufacturer’s instructions.

### Offline high pH reversed peptide fractionation

The TMT-labeled peptides were subjected to high pH reversed-phase peptide fractionation using an Agilent 1290 bioinert high-performance liquid chromatography system (Agilent, Santa Clara, CA, USA) equipped with an analytical column (ZORBAX 300Extend C18 column, 4.6 × 150 mm, 3.5 µm). Solvents A and B contained 15 mM ammonium hydroxide in water and 15 mM ammonium hydroxide in 90% ACN, respectively. The peptides were separated using a 40-min gradient of 5 to 35% ACN at a flow rate of 0.2 mL/min.

Overall, 96 fractions were non-contiguously concatenated into 24 fractions. The fractionated samples were dried using a Speed-Vac and stored at –80 °C until further analysis.

### Liquid chromatography-tandem MS analysis

The peptide samples were analyzed using a Q-Exactive HF-X system (Thermo Fisher Scientific) combined with an Ultimate 3000 RSLC system (Dionex, Sunnyvale, CA, USA) equipped with an EASY nanospray source (Thermo Fisher Scientific), as previously described. The nanoflow liquid chromatography system was configured with two columns: a trap column (1 mm i.d. × 5 mm, 5 µm) and an analytical column (75 µm i.d. × 50 cm, 2 µm, 100 Å). For label-free quantification, peptide samples were separated using a 90-minute gradient of 10 to 30% ACN at 300 nL/min. For the TMT experiments, the peptide samples were separated using a 180-minute gradient of 10–30% ACN at 300 nL/min. The column temperature was maintained at 60 °C with a column heater. The MS1 scan was acquired in the 30–1 650 m/z range with a resolution of 70,000 at 200 m/z. The MS/MS analysis involved high-energy collisional dissociation using a normalized collision energy of 30 and a resolution of 17,500 at 200 m/z. MS/MS was performed in data-dependent acquisition mode to select the top of 15 most abundant precursor ions, with an isolating window of 0.7 m/z for precursor ion selection.

### Data processing for MS analysis

For reporter ion quantification in the TMT-labeled experiments, MS spectra files were processed using Proteome Discoverer version 2.3 with the SEQUEST-HT algorithm against the HUMAN UniProt sequence database (December 2014, 88,757 entries) and common contaminants. The raw spectrum was filtered with a precursor mass size of 350–5000 Da. Trypsin and Lys-C were used as the digestion enzymes for searches involving tryptic peptides. Fully tryptic fragments with up to a maximum of 2 missed cleavages were allowed. The precursor and fragment mass tolerances were set to 20 ppm and 0.02 Da, respectively. Methionine oxidation (15.995 Da) and glutamine and asparagine deamination (0.984 Da) were set as dynamic modifications. Carbamidomethylation of cysteine residues (57.021 Da) and TMT labeling of any N-terminus of the peptide and lysine (229.153 Da) were set as static modifications. The co-isolation threshold was set at 50%. Peptide spectrum matches were validated using a percolator to remove the decoy spectrum at a false discovery rate (FDR) of 1%. The protein confidence criterion was set to an FDR of 1% at the protein level.

MS raw files for the 5/6 Nx rat model were analyzed using the MaxQuant (version 1.6.1.0) environment^26^. The spectra were subjected to a database search against the RAT UniProt sequence database (September 2018; 37 316 entries with 248 common contaminants) using the Andromeda search engine. The precursor mass tolerances were set to 20 and 4.5 ppm for the first and main search, respectively. The fragment mass tolerance for the HCD MS/MS spectra was set to 20 ppm. The enzyme specificity was set to complete tryptic digestion. Peptides with a minimum length of 6 amino acids and up to two missed cleavages were included. Cysteine carbamidomethylation and methionine oxidation were specified as fixed and variable modifications, respectively. The acceptable FDR was set to 1% at the peptide, protein, and modification levels. Protein quantification was performed using intensity-based absolute quantification (iBAQ)^27^. The iBAQ value is obtained by dividing the protein intensities by the number of theoretically observable tryptic peptides between 6 and 30 amino acids^27^. The MS proteomic data have been deposited in the ProteomeXchange Consortium via the PRIDE^28^ partner repository with the dataset identifier PXD043922.

### Quantitative reverse-transcription polymerase chain reaction

Total RNA was extracted from the kidney tissues of 5/6 Nx rats using a RNeasy kit (Qiagen GmbH, Hilden, Germany). The mRNA levels of the target genes, including *Fn1*, *Tufm*, *Gfm1*, *Acadm*, *Sparc*, and *Tagln* (Applied Biosystems, Waltham, MA, USA), were measured by quantitative real-time reverse-transcription polymerase chain reaction (qRT-PCR). qRT-PCR was performed via either Assay-on-Demand TaqMan probes or the SYBR Green method, and an Applied Biosystems PRISM 7500 sequence detection system was used for analysis. The data were normalized to the *Gapdh* level and presented as the fold increase compared to the expression level of RNA isolated from the control group, using the 2^−ΔΔCT^ method. All the experiments were performed in triplicate. The primers used for qRT-PCR are listed in Supplementary Table 2.

### Immunohistochemistry

Immunohistochemistry (IHC) was performed to confirm the establishment of the kidney fibrosis model and to assess TAGLN expression. Paraffin-embedded sections of rat and human kidneys cut into 4-μm-thick slices were deparaffinized and hydrated using xylene and ethanol. Endogenous streptavidin activity was blocked using 3% hydrogen peroxide. Periodic acid Schiff base, Masson’s trichrome, and Sirius red staining (Abcam) were performed to evaluate the degree of renal tubular injury, collagen deposition, and kidney fibrosis. Additionally, superoxide dismutase type 1 (SOD-1; Szabo-Scandic, Vienna, Austria) and cytochrome C (Cyt C; Thermo Fisher Scientific) staining was performed to assess mitochondrial function, and NGAL staining (Santa Cruz Biotechnology, Dallas, TX, USA) was used to evaluate renal tubular injury. The deparaffinized sections were also stained with an anti-TAGLN antibody (Proteintech, Rosemont, IL, USA; Supplementary Table 3).

All the stained sections were counterstained with Mayer’s hematoxylin (Sigma‒Aldrich) and evaluated under a light microscope using a camera with differential interference contrast agent (DFC-295; Leica, Wetzlar, Germany). The expression levels were determined by randomly selecting 10 fields at ×100 original magnifications. All the images were acquired using a Leica DM750 microscope and analyzed using Leica LasX software.

### Western blotting

The protein content of hTECs was extracted using a radioimmunoprecipitation assay buffer containing a Halt protease inhibitor (Pierce, Rockford, IL, USA). The extracted proteins were subsequently aliquoted in equal amounts (30–60 μg) and loaded onto 10% sodium dodecyl sulfate-polyacrylamide gels for electrophoresis. After electrophoresis, the proteins were transferred onto Immobilon FL 0.4 μM polyvinylidene difluoride membranes (Millipore, Bedford, MA, USA), which were probed with antibodies against TAGLN (Abcam), collagen type I alpha 1 chain (COL1A1; Novus Biologicals, Centennial, CO, USA), α-smooth muscle actin (αSMA) (αSMA; Abcam), and β-actin (Sigma‒Aldrich). The probed proteins were detected using chemiluminescence (ECLTM PRN 2106; Amersham Pharmacia Biotech, Buckinghamshire, UK) and gel documentation (Gel Doc 1000 and Multi-Analyst version 1.1; Bio-Rad Laboratories, Hercules, CA, USA) systems. Finally, the Western blotting images were semi-quantified using ImageJ ver. 1.52a (Wayne Rasband, National Institutes of Health, Bethesda, MD, USA).

### Reactive oxygen species (ROS) assay

An OxiSelect Intracellular ROS Kit (Cell Biolabs, San Diego, CA, USA) was used for intracellular ROS measurement. Fibrocellular and acute kidney injury (AKI) models were established in hTECs by treating with rTGF-β (2 ng/mL for 48 hours) and H_2_O_2_ (1 mM for 1 hour), respectively. iTAGLN was administered at concentrations of 0, 0.5, or 1.0 μg/mL simultaneously with each stressor. After treatment, the cells were washed with Hank’s balanced salt solution and incubated with 100 μL of cell-permeable and fluorogenic compound (2′,7′-dichlorodihydrofluorescein diacetate, DCFH-DA)/medium for 30 min at 37 °C. The fluorescence intensity of DCFH-DA was then measured using a microplate fluorometer (Molecular Devices, San Jose, CA, USA) at an excitation wavelength of 485 nm and an emission wavelength of 530 nm.

### Bioinformatic analysis

The protein quantification tables obtained with TMT and label-free quantification were processed using Perseus software^29^. For pairwise comparisons of the TMT datasets, the reporter ion intensities were log_2_-transformed. After normalization using the width adjustment module in Perseus software, pairwise *t* tests were performed using permutation-based FDR with a significance cutoff of 5%. A log_2_-transformed label-free quantification dataset was used to impute missing values for proteins with at least 70% valid values. The data were normalized for statistical analyses. A *t* test was performed to identify proteins showing significant differences in expression (permutation-based FDR of 0.05).

Differentially expressed proteins (DEPs) of hTECs and rat kidneys were subjected to Gene Ontology (GO) analysis using the “enrichR” package in R (version 4.2)^30^. The resulting datasets were processed using in-house R scripts for data visualization with the GOcircle plot in the “GOplot” package^31^. All the GO terms were calculated as z scores using the following formula in the GOplot package:

$${zscore}=\frac{{up}-{down}}{\sqrt{{count}}}\,,$$

where *count* is the number of proteins assigned to a given term, and *up* and *down* are the numbers of proteins assigned as upregulated (log_2_-fold change > 0) or downregulated (log_2_-fold change < 0), respectively, in each comparison.

Functional GO analysis of the common DEPs between hTECs and rat kidneys was performed based on the MSigDB gene sets using gene set enrichment analysis (GSEA; version 4.1.0)^32^. A network model for protein-protein interactions was constructed using the STRING database^33^ and visualized using Cytoscape (version 3.10.0)^34^. For network analysis, importance scores for selecting hub genes were determined with the iGraph package^35^.

### Statistical analysis

The normalized iBAQ scores were compared between the normal and CKD stages using Welch’s *t* test to measure the concentration of TAGLN in the urine of patients with CKD. The urine proteome of patients with CKD was quantified in our previous study^17^. The correlation between kidney tissue TAGLN expression in CKD patients and conventional laboratory findings was tested by Pearson’s correlation analysis.

Statistical analysis was performed using GraphPad Prism 8.3 (GraphPad, Inc.) for validation. The data are expressed as the means ± standard deviations. Pairwise comparisons of multiple groups were performed using the Kruskal-Wallis test with FDR adjustment, and statistical significance was set at *p* < 0.05.

## Results

### Protein profiling scheme related to CKD hTECs and the 5/6 Nx rat model

A schematic diagram of the workflow is illustrated in Fig. 1. MS-based proteomic analysis was performed to identify changes in protein expression during kidney fibrosis.

We selected hTECs as an experimental model as renal tubular epithelial fibrosis is a common terminal manifestation of the progression of kidney fibrosis. Furthermore, we chose the transforming growth factor β (TGF-β) model to target the specific common changes in kidney fibrosis. To induce an appropriate level of fibrosis while avoiding excessive apoptosis, we opted for a 48-hour stimulation period. We selected the Nx model as it reflects the physiological changes observed in CKD patients, regardless of the underlying cause (Supplementary Fig. 1).

### Differential protein abundance in the proteome and the cellular model

**Fig. 2:**
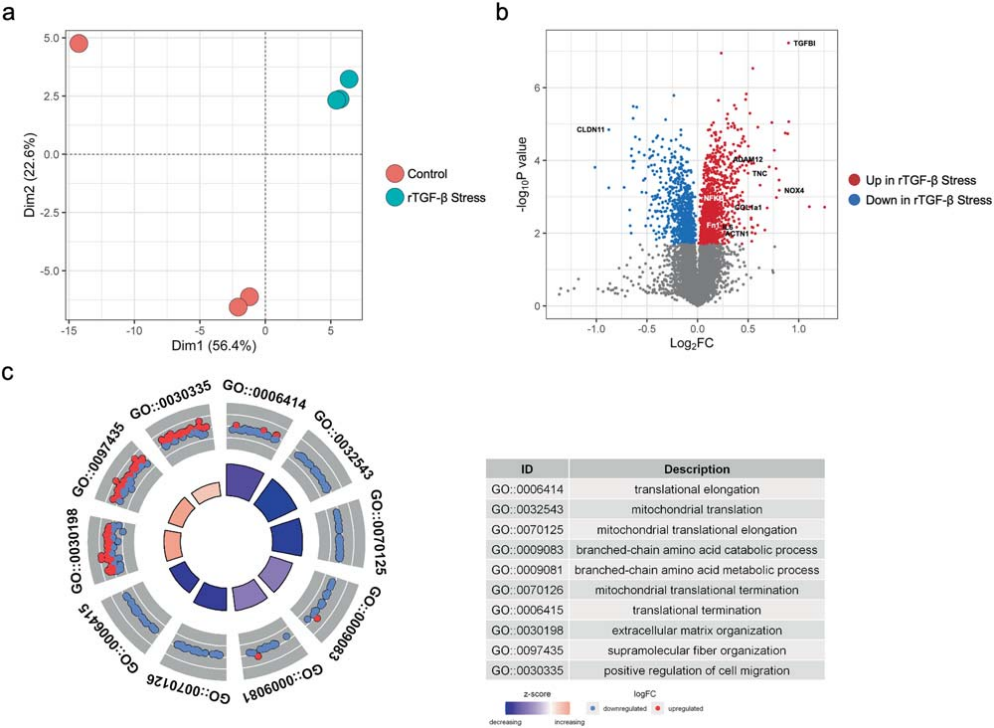
Identification of differentially expressed proteins from the cellular model of chronic kidney disease. **a** Principal component analysis of isobaric-labeled proteome abundance between normal and fibrotic hTECs. Each dot represents the proteome abundance of one sample (*n* = 3). **b** The DEPs for the comparison are represented by a volcano plot. Blue and red indicate significantly downregulated and upregulated proteins, respectively, and black indicates no significant difference. Highly significant differentially expressed proteins are labeled on the volcano plot. **c** Gene Ontology (GO) analysis results for the DEPs. Red and blue dots indicate the log_2_-fold change in each protein that included individual GO terms. hTECs, human primary tubular epithelial cells; DEPs, differentially expressed proteins.

Proteomic profiling of hTECs from the rTGF-β-induced fibrosis and control groups, with three biological replicates each, was conducted using TMT-multiplexed liquid chromatography with tandem MS quantification analysis. Overall, 10,324 proteins were quantified in the TMT-labeled global proteome profile (Supplementary Table 4). The principal components analysis revealed a distinct separation of the overall proteome profiles between the two groups (Fig. 2a). After quantification, we identified 2 100 DEPs (upregulated: 1073 proteins; downregulated: 998 proteins) using a permutation-based FDR of 5% (Fig. 2b and Supplementary Table 5).

Among the DEPs, we identified several proteins associated with kidney injury and organ fibrosis, such as transforming growth factor beta-induced (TGFBI), COL1A1, fibronectin 1 (FN1), actinin alpha 1 (ACTN1), NADPH oxidase 4 (NOX4), nuclear factor kappa B subunit, interleukin 6 (IL-6), tenascin C (TNC), ADAM metallopeptidase domain 12 (ADAM12), lysyl oxidase (LOX), lysyl oxidase-like 3 (LOXL3), and claudin 11 (CLDN11). CLDN11 was downregulated in rTGF-β-treated hTECs, while the remaining proteins were upregulated (Fig. 2b and Supplementary Fig. 2). The RDG-containing protein TGFBI is involved in cell-collagen interactions and is induced by rTGF-β^36^. COL1A1, FN1, and ACTN1 are classical fibrosis markers; NOX4 is a kidney superoxide-producing NADPH oxidase involved in the differentiation of fibroblasts into myofibroblasts and the synthesis of ECM proteins^37^; NFKB and IL-6 are known inflammation markers; TNC promotes fibroblast activation and ECM production^38^; ADAM12 is associated with liver fibrosis^39^; LOX, and LOXL3 accelerate kidney fibrosis via collagen cross-linking^40^.

The significantly downregulated proteins were associated with translational elongation, mitochondrial translation, branched-chain amino acid catabolism, and metabolic processes. In contrast, the upregulated proteins were predominantly involved in ECM organization, supramolecular fiber organization, and positive regulation of cell migration under rTGF-β stress conditions (Fig. 2c and Supplementary Table 6). Proteomic analysis of our fibrotic hTEC model confirmed the observed decline in mitochondrial function and amino acid metabolism and an increase in the ECM and identified several proteins implicated in these processes.

### Differential protein abundance of the proteome in the 5/6 Nx model

**Fig. 3:**
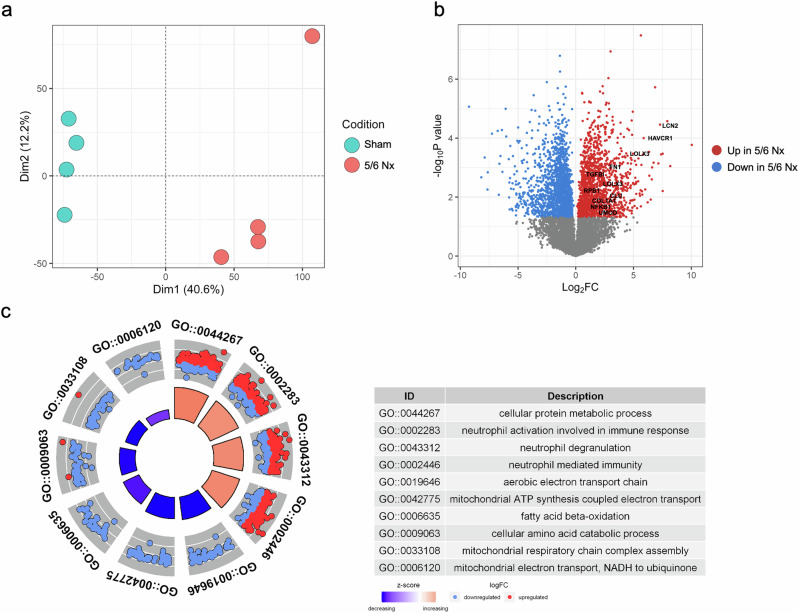
Identification of differentially expressed proteins in a chronic kidney disease animal model. **a** Results of principal component analysis showing the first two dimensions (Dim1 and Dim2) for all samples (sham, *n* = 4; 5/6 nephrectomy [Nx], *n* = 4). **b** Volcano plots represent significantly differentially expressed proteins with log_2_-fold changes plotted with negative log_10_ *p* values for the 5/6 Nx group compared with the sham group. Red and blue dots indicate upregulated and downregulated proteins, respectively. **c** Results of the GO biological process category with all DEPs of sham and 5/6 Nx-operated rats. Red and blue dots indicate the log_2_-fold change of expression levels compared to the sham and 5/6 Nx rat kidney tissues. The z score for each GO term was calculated and displayed with a GOcircle plot. GO, Gene Ontology.

The global proteomic profiles of the kidney tissue in the 5/6 Nx model were investigated using label-free quantification. Overall, 9187 proteins were identified, and distinct clusters were observed between the sham and 5/6 Nx groups (Fig. 3a). Further analysis focused on 7245 quantified proteins (Supplementary Table 7), resulting in the identification of 2835 significant DEPs (1 339 upregulated proteins; 1496 downregulated proteins) between the sham and 5/6 Nx groups (Fig. 3b and Supplementary Table 8) after an FDR adjustment of 5%.

Twelve proteins associated with kidney injury and fibrosis were identified. In the cellular model, six proteins related to kidney fibrosis, namely, TGFBI, FN1, NFKB, TNC, ACTN1, and COL1A1, elevated expression levels. Six other proteins, including lipocalin 2, epidermal growth factor, uromodulin, hepatitis A virus cellular receptor 1, clusterin, and retinol-binding protein 1, were also associated with kidney injury (Supplementary Fig. 3). These proteins are well-known markers of kidney injury, and their expression patterns are consistent with those reported in previous studies^41–47^, supporting their relevance in the context of kidney fibrosis in our proteome expression profiling of 5/6 Nx rats.

We performed functional annotation using GO analysis of the relative expression levels of the DEPs (Fig. 3c). The downregulated proteins were significantly involved in mitochondria-related functions, including the aerobic electron transport chain, mitochondrial adenosine triphosphate synthesis-coupled electron transport, fatty acid beta oxidation, mitochondrial respiratory chain complex assembly, mitochondrial electron transport, and NADH to ubiquinone. Proteins related to neutrophil activity, such as neutrophil activation in the immune response, neutrophil degranulation, and neutrophil-mediated immunity, were significantly upregulated in 5/6 Nx rats (Fig. 3c and Supplementary Table 9). Proteomic analysis of the 5/6 Nx model revealed a reduction in mitochondrial function, which was consistent with the results obtained in the hTEC model. We observed an elevated inflammatory response in this animal model.

### Comprehensive identification of fibrosis-associated changes in protein expression in vitro and in vivo

**Fig. 4:**
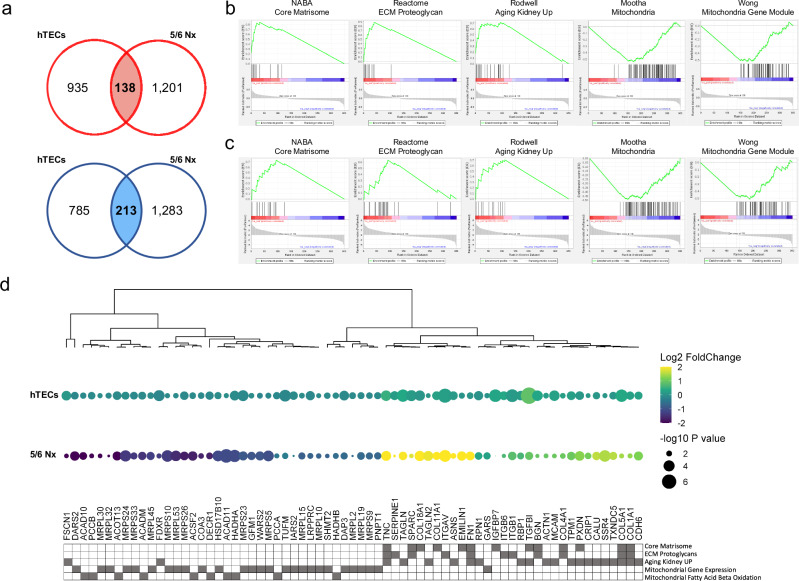
Functional ontology analysis of concurrently regulated differentially expressed proteins. **a** Venn diagram indicating the differentially expressed proteins (DEPs) for comparisons between cellular models and in vivo rat models, particularly each up- and downregulated protein. **b**, **c** Gene set enrichment analysis of the intersection of DEPs identified from hTECs and nephrectomy-operated rats. **d** Dot heat map showing the expression patterns of 69 proteins related to renal and chronic diseases. Hierarchical clustering of proteins was performed using a Euclidean distance matrix of log_2_-fold change of expression levels. The dot size indicates the negative log-transformed *p* value. The lower gray color in the heat maps indicates proteins that induced each GSEA term. GSEA, gene set enrichment analysis.

To understand the comprehensive molecular signatures and pathways relevant to DEP-related kidney fibrosis, we conducted further analyses by focusing on the 351 DEPs that exhibited concordant alterations in expression across both cellular and animal models. Among these proteins, 138 were concurrently upregulated, and 213 were downregulated during kidney fibrosis (Fig. 4a).

We determined the dysfunctional kidney-related biological processes related to CKD using GSEA. As shown in Fig. 4b, c, we identified the activation of several processes and components, including the core matrisome, ECM proteoglycan, and kidney aging, in damaged kidneys, whereas mitochondrial and mitochondrial gene modules were significantly inhibited in both CKD models. When the functional progression of CKD was analyzed, 69 proteins were found to be related to selected biological processes (Supplementary Table 10). When the relative abundance of those 69 proteins was compared using a heat map and categorized matrices for each enrichment term, most of the upregulated proteins in the CKD models were related to the ECM and aged kidneys (Fig. 4d). In contrast, the downregulated proteins were related to mitochondrial gene expression and fatty acid beta-oxidation. Compared with the cellular model, the 5/6 Nx rat model revealed a largely difference in protein abundance between the sham and chronic stress conditions because the hTECs were analyzed using TMT isobaric labeling. The quantified method for TMT labeling shown ratio compression during quantification.

### Protein-protein interaction analysis of fibrosis-associated DEPs

**Fig. 5:**
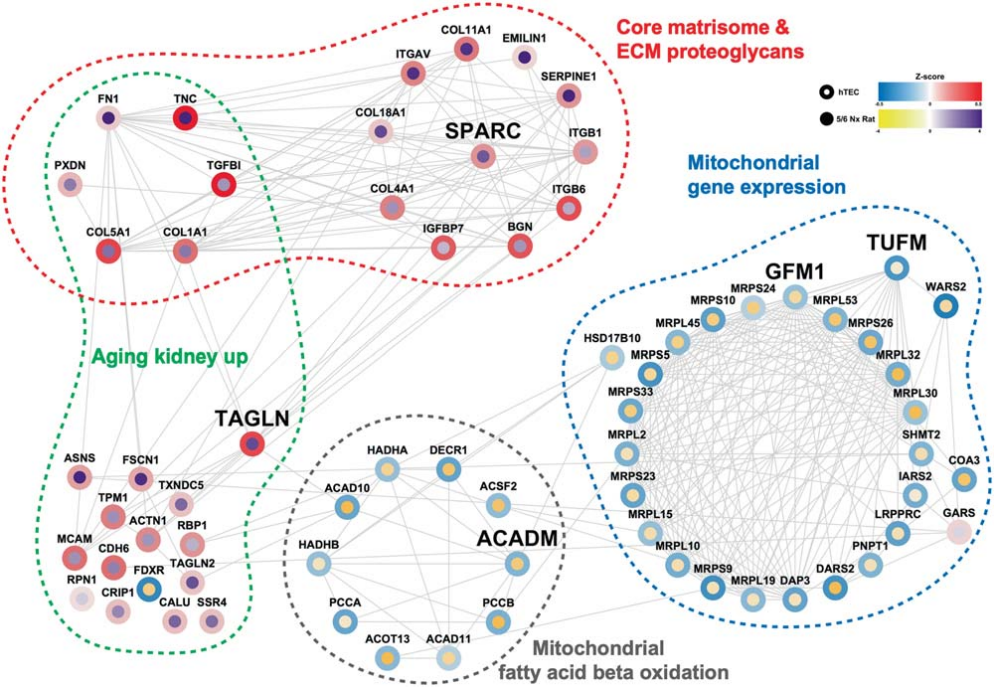
Network analysis of functional annotated differentially expressed proteins. Protein interaction network models related to renal and chronic diseases. The networks were reconstructed for protein-protein interactions from the STRING database and visualized using Cytoscape. The log_2_-fold protein abundance of hTECs in the 5/6 Nx rats is indicated at the border and circle of the node. Each edge represents the protein-protein interactions obtained from the STRING database. hTECs, human primary tubular epithelial cells.

We performed a protein-protein interaction analysis using the STRING database to investigate the interaction network of the 69 crucial fibrosis-associated proteins (Fig. 5). Five proteins that did not interact with other proteins were excluded. As expected, the network analysis revealed that FN1, TGFBI, and TNC, which have been previously implicated in the process of kidney fibrosis, that associated with the ECM proteoglycans and core matrix pathways^8,48^. Furthermore, associations were observed among proteins involved in kidney aging and the fibrosis pathway^4^.

Protein interaction networks were further classified based on biological processes using GSEA, which identified four distinct protein groups. Interaction scores for each biological process were determined using the iGraph network scoring algorithm (Supplementary Table 11). Notably, we focused on five proteins (TAGLN, acyl-CoA dehydrogenase medium chain [ACADM], G elongation factor mitochondrial 1 [GFM1], secreted protein acidic and rich in cysteine [SPARC], and translation elongation factor mitochondrial [TUFM]) with high interaction scores within each biological process after excluding structural proteins, such as fibronectin, integrin, collagen protein, and mitochondrial ribosomal structure subunits. In particular, TAGLN significantly interacts with known fibrosis-related proteins, including FN1 and TPM1^49^, as well as SPARC, ACAD10, biglycan, and melanoma cell adhesion molecules, which are functionally linked to ECM synthesis, fatty acid β-oxidation, collagen fibril assembly, and cell adhesion^50–53^. Therefore, we selected TUFM, GFM1, ACADM, SPARC, and TAGLN as the five signature proteins for further validation.

### Selection of target proteins by comparing the mRNA expression of the five proteins selected from proteomics

**Fig. 6:**
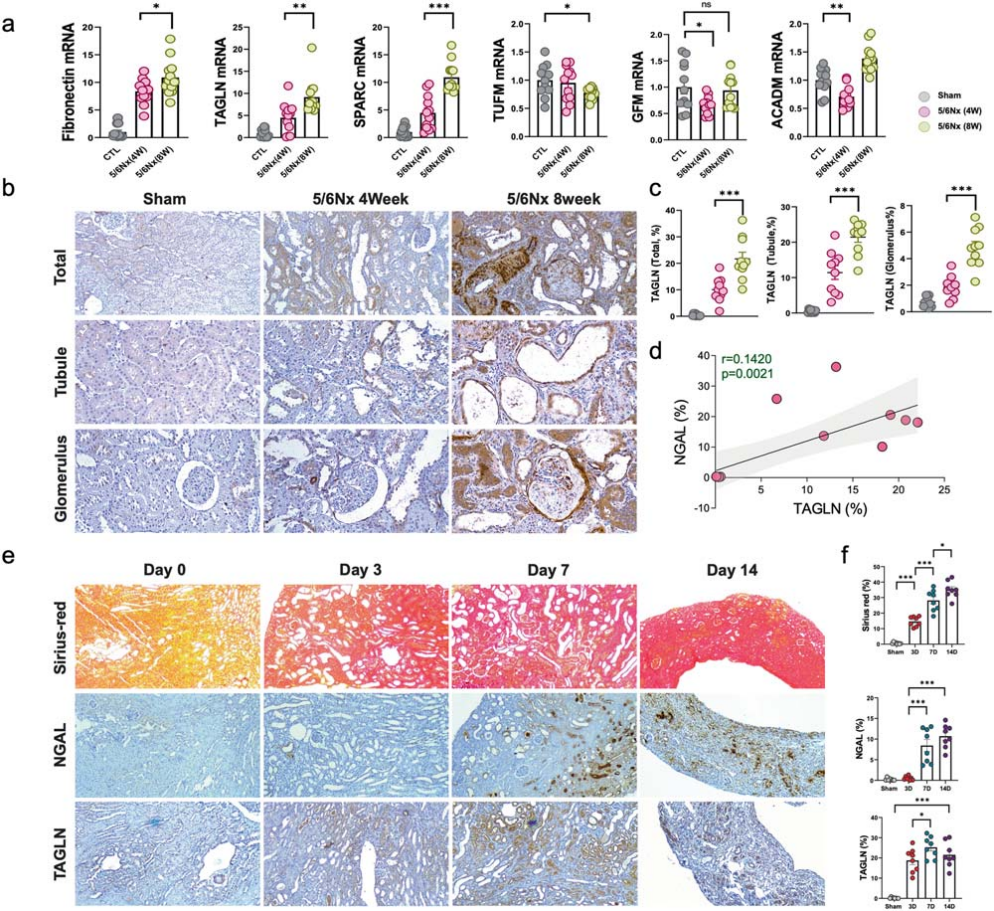
Elevated expression of *Tagln* in two animal models of kidney fibrosis. **a** The mRNA expression of fibronectin and five selected proteins (TAGLN, SPARC, TUFM, GFM1, and ACADM) was measured in the kidney tissue of the 5/6 Nx rat model (n = 12 for each group). **b** Immunohistochemistry (IHC) images of TAGLN in the kidney tissue of sham and 5/6 Nx rats. Kidney tissue was assessed entirely (first row, magnification: ×100) and separately with renal tubules (second row, magnification: ×200) and glomerulus (third row, magnification: ×200). **c** Correlation plot comparing the expression of TAGLN with that of NGAL in the kidney tissue of 5/6 Nx rats at 8 weeks (n = 15). The expression of each protein was assessed according to the mean percentage of positive area per field. **d** Quantification of the TAGLN-positive area among the entire tissue, tubules, and glomerulus (n = 10 for each group). **e** IHC images of Sirius red, NGAL, and TAGLN staining in the UUO mouse model. The original magnification for all the samples was ×100. **f** Quantification of the Sirius red-, NGAL-, and TAGLN-positive areas in the total area (n = 8 for each group). *p < 0.05; **p < 0.005; and ***p < 0.001. UUO, unilateral ureteral obstruction.

The mRNA expression of five selected proteins (TAGLN, TUFM, GFM1, ACADM, and SPARC) was revalidated in the kidney tissue of the 5/6 Nx model used for proteomics. *Fn1* mRNA was used as a positive control. These results were consistent with the proteomic data, which revealed significant and gradual upregulation of *Tagln* and *Sparc* and significant downregulation of *Tufm* at 8 weeks (Fig. 6a). The mRNA expression levels of *Gfm1* and *Acadm*, which decreased according to the proteomic analysis in the 8-week model, were also lower than those in the control group in the 4-week 5/6 Nx model. However, no significant difference was observed for *Gfm*1, and an increase in the expression of *Acadm* (*p* < 0.001) was found in the 8-week model, indicating a lack of reproducibility.

Given the concordance between the mRNA and proteomic data (Supplementary Fig. 4), TAGLN and SPARC were selected as candidate proteins for further validation. While the collagen-binding matricellular protein SPARC was associated with extracellular matrix-related proteins in the interaction network analysis, TAGLN was linked to both aging and mitochondrial processes in addition to the extracellular matrix. Therefore, we prioritized further validation of TAGLN as a protein potentially involved in kidney fibrosis.

### Increased TAGLN expression in the kidney tissue of the 5/6 Nx rat model and the unilateral ureteral obstruction mouse model

The mRNA expression of *Tagln* increased gradually from 4 to 8 weeks in the kidney tissue of 5/6 Nx rats (Fig. 6b). The mean TAGLN staining intensity was consistent with the tissue expression of NGAL (Fig. 6c). The expression of Tagln was observed and compared with each section to investigate whether there was a difference in expression between the glomeruli and renal tubules. The proportion of Tagln-positive areas was greater in the tubular area than in the glomeruli, and the Tagln expression in the glomeruli was primarily concentrated in the periglomerular area (Fig. 6d).

A UUO model was established to evaluate Tagln expression changes during the progression from AKI to CKD. The tissue expression of Ngal, a known biomarker of AKI, significantly increased from day 7, whereas Tagln expression considerably increased from day 3 and remained until day 14 (Ngal: sham 0.22%, day 3 0.51%, *p* value 0.141; Tagln: sham 0.18%, day 3 18.79%, *p* < 0.001). These results suggest that Tagln also has potential as a biomarker for AKI and early CKD progression (Fig. 6e, f).

### Validation of TAGLN expression in kidney biopsy and urine samples from patients with CKD

**Fig. 7:**
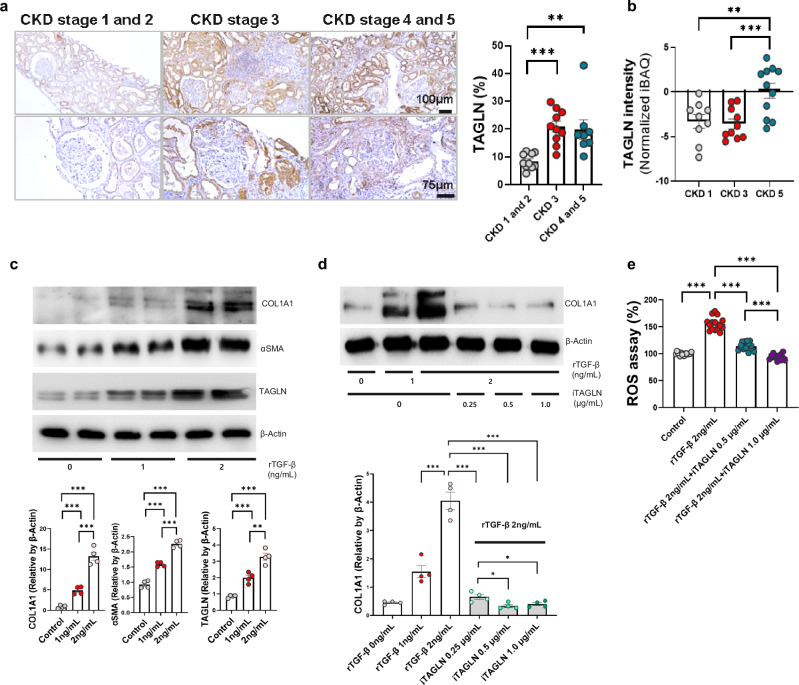
Elevated expression of TAGLN in patients with chronic kidney disease and amelioration of fibrosis in hTECs through TAGLN inhibition. **a** IHC images of TALGN staining in consecutive patients with CKD stages 1 and 2 (n = 10), 3 (n = 10), and 4 and 5 (n = 8) and quantification of the TAGLN-positive area in the total field on the right side. The magnifications in the first and second rows were ×100 and ×200, respectively. **b** Comparison of urinary TAGLN expression levels between the normal and CKD groups. The urinary TAGLN level was measured using normalized iBAQ (CKD stage 1, n = 9; CKD stage 3, n = 10; CKD stage 5, n = 11). **c** Western blotting (top) and semi-quantification (down) results after 48 h of rTGF-β stress (n = 4 for each group). **d** Western blotting (top) and semi-quantification (down) results after 48 h of rTGF-β stress with or without iTAGLN treatment (n = 4 for each group; **p < 0.005; ***p < 0.001). **e** ROS assay after 48 h of rTGF-β stress with or without iTAGLN treatment in hTECs (n = 16). CKD, chronic kidney disease; hTECs, human primary tubular epithelial cells; iBAQ, intensity-based absolute quantification; IHC, immunohistochemical; ROS, reactive oxygen species; rTGF-β, recombinant transforming growth factor β; and TAGLN, transgelin.

The tissue expression of TAGLN was significantly greater in patients with moderate (mean ± standard error of the mean; CKD stages 1 and 2, 8.50 ± 0.87%; CKD stage 3, 20.86%; *p* < 0.001) to advanced CKD (CKD stages 1 and 2; CKD stages 4 and 5, 19.85 ± 3.50%; *p* = 0.003) than in those with early CKD (Fig. 7a). However, no significant differences were observed between patients with moderate (stage 3 CKD) and advanced (stage 4 and 5 CKD, *p* = 0.794) disease. Additionally, the expression level of TAGLN in kidney tissue was significantly correlated with conventional serum renal function indicators, including the eGFR (Pearson’s correlation coefficient (R) = −0.60, *p* = 0.001) and blood urea nitrogen level (R = 0.36, *p* = 0.041; Supplementary Fig. 5).

The expression of TAGLN in urine samples was significantly increase in patients with advanced CKD than in those with early CKD (CKD stage 1, −3.30 ± 0.76; CKD stage 5, 0.12 ± 0.84; *p* = 0.009) and moderate CKD (CKD stage 3, −3.58 ± 0.55; CKD stage 5; *p* = 0.002; Fig. 7b). However, no difference in TAGLN staining intensity was observed between patients with CKD stages 1 and 3 (*p* = 0.762).

The results from patients with CKD indicated that the local expression of TAGLN increased and remained elevated from the early stages of CKD in kidney tissue. As CKD progresses, TAGLN could also be detected in urine, suggesting that TAGLN is a promising noninvasive biomarker for kidney fibrosis.

### TAGLN blockade ameliorates the progression of kidney fibrosis

To determine whether blocking TAGLN has therapeutic potential in the progression of kidney fibrosis, we inhibited TAGLN in fibrosis-induced hTECs. When hTECs were subjected to 48 h of TGF-β stress to induce fibrosis, the expression levels of TAGLN, COL1A1, and αSMA increased in a concentration-dependent manner in response to rTGF-β (Fig. 7c). A significant reduction in kidney fibrosis was observed with both 0.5 μg/mL and 1.0 μg/mL iTAGLN (2 ng/mL rTGF-β: 4.30 ± 0.31; 2 ng/mL rTGF-β + 0.5 μg/mL iTAGLN: 0.34 ± 0.05 vs. 2 ng/mL rTGF-β, *p* < 0.001; 2 ng/mL rTGF-β + 1.0 μg/mL iTAGLN 0.40 ± 0.05 vs. 2 ng/mL rTGF-β, *p* < 0.001). However, no significant difference was observed between 0.5 μg/mL and 1.0 μg/mL iTAGLN (*p* = 0.427, Fig. 7d). Despite its lower potency compared to the higher doses, a significant inhibitory effect on kidney fibrosis was observed at 0.25 μg/mL (2 ng/mL rTGF-β + 0.25 μg/mL iTAGLN: 0.53 ± 0.08, *p* < 0.001). These findings suggest that iTAGLN can significantly ameliorate kidney fibrosis at relatively low concentrations.

We hypothesized that TAGLN activates mitochondrial dysfunction to regulate ROS levels via actin cytoskeleton dynamics^54^. To test our hypothesis, we blocked TAGLN in hTECs with rTGF-β-induced kidney fibrosis and observed an increase in ROS levels. The increase in ROS levels induced by rTGF-β was mitigated in a dose-dependent manner by iTAGLN treatment (Fig. 7e). Additionally, we observed that the increase in ROS levels caused by H_2_O_2_ stress was also reduced by iTAGLN (Supplementary Fig. 6). These findings suggest that blocking TAGLN can prevent kidney damage caused by elevated ROS levels not only from TGF-β but also from various other factors.

## Discussion

Our multidisciplinary study highlighted the potential use of TAGLN as a prognostic biomarker and therapeutic target for kidney fibrosis. We elucidated global proteomic alterations via MS-based proteome analysis in both human-derived cellular and animal models of CKD. To investigate fibrosis-associated proteins across the human-derived cellular model and animal kidney tissues afflicted with fibrosis, we observed concordant expression patterns between the DEPs in our models. Through global proteomic profiling, we identified 315 proteins that were concurrently altered during kidney fibrosis. GSEA refined our selection to 69 proteins associated with the ECM, aging, and mitochondrial functions. In the network analysis of these 69 DEPs, TAGLN, GFM1, TURM SPARC, and ACADM emerged as key proteins potentially linked to kidney fibrosis markers. Notably, TAGLN demonstrated the strongest interaction with established fibrosis-related proteins from multiple human-derived samples, suggesting its potential as a noninvasive biomarker and therapeutic target for kidney fibrosis.

In several studies of UUO models, TAGLN upregulation has been observed in the interstitial space and periglomerular cells, particularly in activated fibroblasts expressing αSMA^55^. Another previous study using an anti-glomerular basement membrane rat model reported increased *Tagln* expression in glomerular epithelial cells during the early phase and subsequently in peritubular interstitial cells during the late phase^56^. TAGLN was also upregulated during the podocyte injury process in specific studies of patients with glomerulonephritis^3^. In our study, we used hTECs as a cellular model and observed an increase in TAGLN expression during the progression of kidney fibrosis induced by TGF-β treatment. We observed a predominant increase in Tagln expression in the renal tubules of 5/6 Nx rats. These findings suggest that TAGLN expression primarily increases in renal tubular cells as kidney fibrosis progresses. Moreover, Tagln expression was increased in the glomerular area in our study, a finding supported by other research, suggesting a potential role for Tagln in glomerular fibrosis.

TAGLN is expressed predominantly in smooth muscle cells^57^, the lung mesenchyme, bronchial epithelial cells, and immune cells^58^. Previous studies have focused on the role of TAGLN in pulmonary fibrosis models as well as in the lung tissue of patients with idiopathic pulmonary fibrosis^59,60^. These studies revealed that TGF-β/SMAD3 upregulates TAGLN by binding to its promoter^59,61^, leading to pulmonary fibrosis through the mitochondria-mediated apoptotic pathway in a p53-dependent manner^3^. In addition, the TAGLN homolog in yeast (Scp1) is known to regulate the dynamics of the actin cytoskeleton, which increases ROS via mitochondrial depolarizations^62^. We found that TAGLN was associated with not only well-known fibrotic proteins such as FN1 and COL11A1 but also mitochondria-regulated proteins during kidney fibrosis. Given these mechanisms, we suspect that TAGLN may also contribute to kidney fibrosis by affecting mitochondrial dysfunction.

In our cellular model, blocking TAGLN ameliorated the fibrosis of hTECs, indicating its potential as a treatment for kidney fibrosis. Although the mechanism of action requires further exploration, our findings indicate that TAGLN inhibition can reduce TGFB-SMAD-induced mitochondrial damage, leading to a therapeutic effect. Overall, given the positive correlation between TAGLN expression and CKD progression and the reduction in kidney fibrosis upon TAGLN blockade, even at low doses, TAGLN may serve as a biomarker and treatment approach for CKD. Additionally, our H_2_O_2_-induced cellular model suggested that the utility of TAGLN could be extended to acute kidney injury (Supplementary Fig. 6).

Our research currently has several limitations. We used hTECs as a cellular model, and tissue validation revealed increased TAGLN expression in both glomerular and tubular cells. These findings necessitate further validation in glomerular cells. We focused primarily on comparing the normal and final fibrotic stages, which might have limited our investigation of potential changes that could occur during the intermediate stages of fibrosis. However, further in-depth mechanistic studies on TAGLN, ROS stress, and mitochondrial dysfunction are needed. Finally, although we explored the potential use of TAGLN as a non-invasive biomarker in various samples from CKD patients and its potential as a therapeutic target for kidney fibrosis through cellular studies, further validation is needed.

However, our research has consistently confirmed through in-depth, extensive exploration that TAGLN expression continuously increases under various conditions of kidney fibrosis in both cellular and animal models, as well as in multiple samples from CKD patients. Furthermore, TAGLN, whose molecular mechanism was previously unknown, is closely connected with mitochondrial-related genes, associated with cellular ROS levels, and correlated with well-known markers of kidney fibrosis. Although further validation in a large cohort is necessary, the potential use of TAGLN as a biomarker for kidney fibrosis has been reported in Chronic Renal Insufficiency Cohort (CRIC) study that used human plasma proteome data. The CRIC study^63^ demonstrated that elevated TAGLN levels were associated with an increased risk of adverse renal outcomes over a 10-year period, which expands the utility of the TAGLN as a prognostic biomarker for CKD.

In conclusion, we investigated proteomic alterations in kidney fibrosis from human-derived cellular and animal models via high-throughput LC-MS/MS proteome profiling. Our analysis revealed that the expression of TAGLN during kidney fibrosis was positively correlated with ROS levels. To demonstrate the expression of TAGLN according to the CKD stage, we investigated TAGLN expression in renal tissues from animal models to support the possibility of its application as a CKD biomarker. This result was also reproduced in kidney tissue and urine samples from CKD patients. Furthermore, we demonstrated that blocking TAGLN in a fibrotic cellular model of hTECs significantly inhibited kidney fibrosis, which suggested the therapeutic potential of TAGLN.

## Supplementary information

Supplementary Information

Supplementary Table 1

Supplementary Table 2

Supplementary Table 3

Supplementary Table 4

Supplementary Table 5

Supplementary Table 6

Supplementary Table 7

Supplementary Table 8

Supplementary Table 9

Supplementary Table 10

Supplementary Table 11

## Data Availability

MS-based proteomics data of all the identified peptides and the protein list have been deposited in the ProteomeXchance Consortium (http://proteomecentral.proteomexchange.org) via the PRIDE partner repository: dataset identifier PXD043922.

## References

[1] Zhou, D. & Liu, Y. Renal fibrosis in 2015: understanding the mechanisms of kidney fibrosis. *Nat. Rev. Nephrol.* **12**, 68–70, 10.1038/nrneph.2015.215 (2016). 26714578 10.1038/nrneph.2015.215 PMC4868356

[2] Tomasek, J. J., Gabbiani, G., Hinz, B., Chaponnier, C. & Brown, R. A. Myofibroblasts and mechano-regulation of connective tissue remodelling. *Nat. Rev. Mol. Cell Biol.* **3**, 349–363, 10.1038/nrm809 (2002). 11988769 10.1038/nrm809

[3] Panizo, S. et al. Fibrosis in chronic kidney disease: pathogenesis and consequences. *Int. J. Mol. Sci.* **22**. 10.3390/ijms22010408 (2021). 10.3390/ijms22010408 PMC7795409 33401711

[4] Fang, Y. et al. The ageing kidney: molecular mechanisms and clinical implications. *Ageing Res. Rev.* **63**, 101151, 10.1016/j.arr.2020.101151 (2020). 32835891 10.1016/j.arr.2020.101151 PMC7595250

[5] Ovadya, Y. & Krizhanovsky, V. A new Twist in kidney fibrosis. *Nat. Med.* **21**, 975–977, 10.1038/nm.3938 (2015). 26340117 10.1038/nm.3938

[6] Bhatia, D., Capili, A. & Choi, M. E. Mitochondrial dysfunction in kidney injury, inflammation, and disease: potential therapeutic approaches. *Kidney Res. Clin. Pract.* **39**, 244–258, 10.23876/j.krcp.20.082 (2020). 32868492 10.23876/j.krcp.20.082 PMC7530368

[7] Ning, B. et al. Calcium signaling mediates cell death and crosstalk with autophagy in kidney disease. *Cells* **10**. 10.3390/cells10113204 (2021). 10.3390/cells10113204 PMC8622220 34831428

[8] Huang, R., Fu, P. & Ma, L. Kidney fibrosis: from mechanisms to therapeutic medicines. *Signal Transduct. Target Ther.* **8**, 129, 10.1038/s41392-023-01379-7 (2023). 36932062 10.1038/s41392-023-01379-7 PMC10023808

[9] Lousa, I. et al. New potential biomarkers for chronic kidney disease management-a review of the literature. *Int. J. Mol. Sci.* **22**. 10.3390/ijms22010043 (2020). 10.3390/ijms22010043 PMC7793089 33375198

[10] Barinotti, A. et al. Serum biomarkers of renal fibrosis: a systematic review. *Int. J. Mol. Sci.* **23**. 10.3390/ijms232214139 (2022). 10.3390/ijms232214139 PMC9697720 36430625

[11] Schmidt, I. M. et al. Cadherin-11, Sparc-related modular calcium binding protein-2, and Pigment epithelium-derived factor are promising non-invasive biomarkers of kidney fibrosis. *Kidney Int.* **100**, 672–683, 10.1016/j.kint.2021.04.037 (2021). 34051265 10.1016/j.kint.2021.04.037 PMC8384690

[12] Catanese, L. et al. Recent advances in urinary peptide and proteomic biomarkers in chronic kidney disease: a systematic review. *Int. J. Mol. Sci.* **24**. 10.3390/ijms24119156 (2023). 10.3390/ijms24119156 PMC10252389 37298105

[13] Zhou, D. & Liu, Y. Understanding the mechanisms of kidney fibrosis. *Nat. Rev. Nephrol.* **12**, 68–70, 10.1038/nrneph.2015.215 (2016). 26714578 10.1038/nrneph.2015.215 PMC4868356

[14] Rinschen, M. M. & Saez-Rodriguez, J. The tissue proteome in the multi-omic landscape of kidney disease. *Nat. Rev. Nephrol.* **17**, 205–219, 10.1038/s41581-020-00348-5 (2021). 33028957 10.1038/s41581-020-00348-5

[15] Byron, S. A., Van Keuren-Jensen, K. R., Engelthaler, D. M., Carpten, J. D. & Craig, D. W. Translating RNA sequencing into clinical diagnostics: opportunities and challenges. *Nat. Rev. Genet* **17**, 257–271, 10.1038/nrg.2016.10 (2016). 26996076 10.1038/nrg.2016.10 PMC7097555

[16] Zhang, B. et al. Clinical potential of mass spectrometry-based proteogenomics. *Nat. Rev. Clin. Oncol.* **16**, 256–268, 10.1038/s41571-018-0135-7 (2019). 30487530 10.1038/s41571-018-0135-7 PMC6448780

[17] Kim, J. E. et al. Multisample mass spectrometry-based approach for discovering injury markers in chronic kidney disease. *Mol. Cell Proteom.* **20**, 100037, 10.1074/mcp.RA120.002159 (2021). 10.1074/mcp.RA120.002159 PMC7950200 33453410

[18] Moon, J. J. et al. Inhibiting transglutaminase 2 mediates kidney fibrosis via anti-apoptosis. *Biomedicines* **10**. 10.3390/biomedicines10061345 (2022). 10.3390/biomedicines10061345 PMC9220123 35740367

[19] Bae, E. et al. Renoprotective effect of KLF2 on glomerular endothelial dysfunction in hypertensive nephropathy. *Cells* **11**. 10.3390/cells11050762 (2022). 10.3390/cells11050762 PMC8909753 35269384

[20] Ryu, S. et al. Siglec-F-expressing neutrophils are essential for creating a profibrotic microenvironment in renal fibrosis. *J. Clin. Invest.* **132**. 10.1172/JCI156876 (2022). 10.1172/JCI156876 PMC9197522 35482420

[21] Han, D., Jin, J., Woo, J., Min, H. & Kim, Y. Proteomic analysis of mouse astrocytes and their secretome by a combination of FASP and StageTip-based, high pH, reversed-phase fractionation. *Proteomics* **14**, 1604–1609, 10.1002/pmic.201300495 (2014). 24753479 10.1002/pmic.201300495

[22] Han, D. et al. In-depth proteomic analysis of mouse microglia using a combination of FASP and StageTip-based, high pH, reversed-phase fractionation. *Proteomics* **13**, 2984–2988, 10.1002/pmic.201300091 (2013). 23943505 10.1002/pmic.201300091

[23] Wisniewski, J. R. & Gaugaz, F. Z. Fast and sensitive total protein and Peptide assays for proteomic analysis. *Anal. Chem.* **87**, 4110–4116, 10.1021/ac504689z (2015). 25837572 10.1021/ac504689z

[24] Kong, S. H. et al. In-depth proteomic signature of parathyroid carcinoma. *Eur. J. Endocrinol.* **188**, 385–394, 10.1093/ejendo/lvad046 (2023). 36995894 10.1093/ejendo/lvad046

[25] Kim, J. Y. et al. Reconstruction of pathway modification induced by nicotinamide using multi-omic network analyses in triple negative breast cancer. *Sci. Rep.* **7**, 3466, 10.1038/s41598-017-03322-7 (2017). 28615672 10.1038/s41598-017-03322-7 PMC5471278

[26] Tyanova, S., Temu, T. & Cox, J. The MaxQuant computational platform for mass spectrometry-based shotgun proteomics. *Nat. Protoc.* **11**, 2301–2319, 10.1038/nprot.2016.136 (2016). 27809316 10.1038/nprot.2016.136

[27] Schwanhausser, B. et al. Global quantification of mammalian gene expression control. *Nature* **473**, 337–342, 10.1038/nature10098 (2011). 21593866 10.1038/nature10098

[28] Perez-Riverol, Y. et al. The PRIDE database resources in 2022: a hub for mass spectrometry-based proteomics evidences. *Nucleic Acids Res.* **50**, D543–D552, 10.1093/nar/gkab1038 (2022). 34723319 10.1093/nar/gkab1038 PMC8728295

[29] Tyanova, S. et al. The Perseus computational platform for comprehensive analysis of (prote)omics data. *Nat. Methods* **13**, 731–740, 10.1038/nmeth.3901 (2016). 27348712 10.1038/nmeth.3901

[30] Chen, E. Y. et al. Enrichr: interactive and collaborative HTML5 gene list enrichment analysis tool. *BMC Bioinforma.* **14**, 128, 10.1186/1471-2105-14-128 (2013). 10.1186/1471-2105-14-128 PMC3637064 23586463

[31] Walter, W., Sanchez-Cabo, F. & Ricote, M. GOplot: an R package for visually combining expression data with functional analysis. *Bioinformatics* **31**, 2912–2914, 10.1093/bioinformatics/btv300 (2015). 25964631 10.1093/bioinformatics/btv300

[32] Subramanian, A. et al. Gene set enrichment analysis: a knowledge-based approach for interpreting genome-wide expression profiles. *Proc. Natl. Acad. Sci. USA* **102**, 15545–15550, 10.1073/pnas.0506580102 (2005). 16199517 10.1073/pnas.0506580102 PMC1239896

[33] Tan, S. & Chao, R. An exploration of osteosarcoma metastasis diagnostic markers based on tumor-associated neutrophils. *Discov. Med.* **35**, 300–311, 10.24976/Discov.Med.202335176.31 (2023). 37272097 10.24976/Discov.Med.202335176.31

[34] Shannon, P. et al. Cytoscape: a software environment for integrated models of biomolecular interaction networks. *Genome Res.* **13**, 2498–2504, 10.1101/gr.1239303 (2003). 14597658 10.1101/gr.1239303 PMC403769

[35] Csardi, G. & Nepusz, T. The igraph software package for complex network research. *InterJ. Complex Syst.* **1695**, 1–9 (2006).

[36] LeBaron, R. G. et al. Beta IG-H3, a novel secretory protein inducible by transforming growth factor-beta, is present in normal skin and promotes the adhesion and spreading of dermal fibroblasts in vitro. *J. Invest. Dermatol.* **104**, 844–849, 10.1111/1523-1747.ep12607024 (1995). 7738366 10.1111/1523-1747.ep12607024

[37] Jiang, F., Liu, G. S., Dusting, G. J. & Chan, E. C. NADPH oxidase-dependent redox signaling in TGF-beta-mediated fibrotic responses. *Redox Biol.* **2**, 267–272, 10.1016/j.redox.2014.01.012 (2014). 24494202 10.1016/j.redox.2014.01.012 PMC3909817

[38] Bhattacharyya, S. et al. Tenascin-C drives persistence of organ fibrosis. *Nat. Commun.* **7**, 11703, 10.1038/ncomms11703 (2016). 27256716 10.1038/ncomms11703 PMC4895803

[39] Du, S. et al. ADAM12 is an independent predictor of poor prognosis in liver cancer. *Sci. Rep.* **12**, 6634, 10.1038/s41598-022-10608-y (2022). 35459884 10.1038/s41598-022-10608-y PMC9033838

[40] Zhang, X. et al. Lysyl oxidase promotes renal fibrosis via accelerating collagen cross-link driving by beta-arrestin/ERK/STAT3 pathway. *FASEB J.* **36**, e22427, 10.1096/fj.202200573R (2022). 35792886 10.1096/fj.202200573R PMC9544652

[41] Sjaarda, J. et al. Blood HER2 and uromodulin as causal mediators of CKD. *J. Am. Soc. Nephrol.* **29**, 1326–1335, 10.1681/ASN.2017070812 (2018). 29511113 10.1681/ASN.2017070812 PMC5875953

[42] Bolignano, D. et al. Neutrophil gelatinase-associated lipocalin (NGAL) and progression of chronic kidney disease. *Clin. J. Am. Soc. Nephrol.* **4**, 337–344, 10.2215/CJN.03530708 (2009). 19176795 10.2215/CJN.03530708 PMC2637601

[43] Lv, L. et al. Serum uromodulin and progression of kidney disease in patients with chronic kidney disease. *J. Transl. Med.* **16**, 316, 10.1186/s12967-018-1693-2 (2018). 30454063 10.1186/s12967-018-1693-2 PMC6245763

[44] Klein, J., Bascands, J. L., Buffin-Meyer, B. & Schanstra, J. P. Epidermal growth factor and kidney disease: a long-lasting story. *Kidney Int.* **89**, 985–987, 10.1016/j.kint.2016.02.020 (2016). 27083276 10.1016/j.kint.2016.02.020

[45] Nakamura, J. et al. Myofibroblasts acquire retinoic acid-producing ability during fibroblast-to-myofibroblast transition following kidney injury. *Kidney Int.* **95**, 526–539, 10.1016/j.kint.2018.10.017 (2019). 30661714 10.1016/j.kint.2018.10.017

[46] Guo, J. et al. Relationship of clusterin with renal inflammation and fibrosis after the recovery phase of ischemia-reperfusion injury. *BMC Nephrol.* **17**, 133, 10.1186/s12882-016-0348-x (2016). 27649757 10.1186/s12882-016-0348-x PMC5028988

[47] Nakagawa, S. et al. Molecular markers of tubulointerstitial fibrosis and tubular cell damage in patients with chronic kidney disease. *PLoS One* **10**, e0136994, 10.1371/journal.pone.0136994 (2015). 26317775 10.1371/journal.pone.0136994 PMC4552842

[48] Massague, J. TGFbeta signalling in context. *Nat. Rev. Mol. Cell Biol.* **13**, 616–630, 10.1038/nrm3434 (2012). 22992590 10.1038/nrm3434 PMC4027049

[49] Huang, H. et al. The MicroRNA MiR-29c alleviates renal fibrosis via TPM1-mediated suppression of the Wnt/beta-Catenin pathway. *Front Physiol.* **11**, 331, 10.3389/fphys.2020.00331 (2020). 32346368 10.3389/fphys.2020.00331 PMC7171049

[50] Wang, Z. et al. CD146, from a melanoma cell adhesion molecule to a signaling receptor. *Signal Transduct. Target Ther.* **5**, 148, 10.1038/s41392-020-00259-8 (2020). 32782280 10.1038/s41392-020-00259-8 PMC7421905

[51] Di Lullo, G. A., Sweeney, S. M., Korkko, J., Ala-Kokko, L. & San Antonio, J. D. Mapping the ligand-binding sites and disease-associated mutations on the most abundant protein in the human, type I collagen. *J. Biol. Chem.* **277**, 4223–4231, 10.1074/jbc.M110709200 (2002). 11704682 10.1074/jbc.M110709200

[52] Ye, X. et al. Cloning and characterization of a human cDNA ACAD10 mapped to chromosome 12q24.1. *Mol. Biol. Rep.* **31**, 191–195, 10.1023/b:mole.0000043622.57408.6b (2004). 15560374 10.1023/b:mole.0000043622.57408.6b

[53] Bradshaw, A. D. The role of SPARC in extracellular matrix assembly. *J. Cell Commun. Signal* **3**, 239–246, 10.1007/s12079-009-0062-6 (2009). 19798598 10.1007/s12079-009-0062-6 PMC2778582

[54] Hausser, J., Mayo, A., Keren, L. & Alon, U. Central dogma rates and the trade-off between precision and economy in gene expression. *Nat. Commun.* **10**, 68, 10.1038/s41467-018-07391-8 (2019). 30622246 10.1038/s41467-018-07391-8 PMC6325141

[55] Zhang, J. C. et al. SM22beta encodes a lineage-restricted cytoskeletal protein with a unique developmentally regulated pattern of expression. *Mech. Dev.* **115**, 161–166, 10.1016/s0925-4773(02)00088-6 (2002). 12049783 10.1016/s0925-4773(02)00088-6

[56] Yin, L. M., Ulloa, L. & Yang, Y. Q. Transgelin-2: biochemical and clinical implications in cancer and asthma. *Trends Biochem Sci.* **44**, 885–896, 10.1016/j.tibs.2019.05.004 (2019). 31256982 10.1016/j.tibs.2019.05.004 PMC7023894

[57] Yu, H. et al. Transgelin is a direct target of TGF-beta/Smad3-dependent epithelial cell migration in lung fibrosis. *FASEB J.* **22**, 1778–1789, 10.1096/fj.07-083857 (2008). 18245174 10.1096/fj.07-083857

[58] Cecchini, M. J., Hosein, K., Howlett, C. J., Joseph, M. & Mura, M. Comprehensive gene expression profiling identifies distinct and overlapping transcriptional profiles in non-specific interstitial pneumonia and idiopathic pulmonary fibrosis. *Respir. Res.* **19**, 153, 10.1186/s12931-018-0857-1 (2018). 30111332 10.1186/s12931-018-0857-1 PMC6094889

[59] Zhang, R. et al. Transgelin as a therapeutic target to prevent hypoxic pulmonary hypertension. *Am. J. Physiol. Lung Cell Mol. Physiol.* **306**, L574–L583, 10.1152/ajplung.00327.2013 (2014). 24464808 10.1152/ajplung.00327.2013

[60] Karagianni, F. et al. Transgelin up-regulation in obstructive nephropathy. *PLoS One* **8**, e66887, 10.1371/journal.pone.0066887 (2013). 23840546 10.1371/journal.pone.0066887 PMC3694161

[61] Sakamaki, Y. et al. Injured kidney cells express SM22alpha (transgelin): Unique features distinct from alpha-smooth muscle actin (alphaSMA). *Nephrology (Carlton)* **16**, 211–218, 10.1111/j.1440-1797.2010.01322.x (2011). 21272134 10.1111/j.1440-1797.2010.01322.x

[62] Gourlay, C. W., Carpp, L. N., Timpson, P., Winder, S. J. & Ayscough, K. R. A role for the actin cytoskeleton in cell death and aging in yeast. *J. Cell Biol.* **164**, 803–809, 10.1083/jcb.200310148 (2004). 15024029 10.1083/jcb.200310148 PMC2172293

[63] Dubin, R. F. et al. Proteomics of CKD progression in the chronic renal insufficiency cohort. *Nat Commun* **14**, 6340, 10.1038/s41467-023-41642-7 (2023). 37816758 10.1038/s41467-023-41642-7 PMC10564759

