# Supplementary material for: Unveiling the role of transgelin as a prognostic and therapeutic target in kidney fibrosis via a proteomic approach
Source: Exp Mol Med. 2024 Oct 7;56(10):2296–308. doi: 10.1038/s12276-024-01319-7 (PMC11542076; doi:10.1038/s12276-024-01319-7)

Supplementary Information

Unveiling the Role of Transgelin as a Prognostic and Therapeutic Target in Kidney Fibrosis via a Proteomic Approach

Soie Kwon[1,2] ¶, Seongmin Cheon[3,4] ¶, Kyu-Hong Kim[5, 6], Areum Seo[5], Eunjin Bae[7], Jae Wook Lee[8], Ran-Hui Cha[9], Jin Ho Hwang[1,10], Yong Chul Kim[11,12], Dong Ki Kim[11, 12], Yon Su Kim[5, 11, 12, 14], Dohyun Han[3,13]* and Seung-Hee Yang[5,14]*

[1]Department of Internal Medicine, Chung-Ang University Hospital, Seoul, Korea

[2]Department of Clinical Medical Sciences, Seoul National University, Seoul, Korea

[3]Proteomics Core Facility, Biomedical Research Institute, Seoul National University Hospital, Seoul, Korea

[4]School of Biological Sciences and Technology, Chonnam National University, Gwangju, Korea

[5]Kidney Research Institute, Seoul National University College of Medicine, Seoul, Korea

[6]Department of Biomedical Sciences, College of Medicine, Seoul National University, Seoul, Korea

[7]Department of Internal Medicine, Gyeongsang National University College of Medicine, Gyeongsang University Changwon Hospital, Gyeongsang, Korea

[8]Nephrology Clinic, National Cancer Center of Korea, Seoul, Korea

[9]Department of Internal Medicine, National Medical Center, Seoul, Korea

[10]Department of Internal Medicine, College of Medicine, Chung-Ang University, Seoul, Korea

[11]Department of Internal Medicine, Seoul National University Hospital, Seoul, Korea

[12]Department of Internal Medicine, Seoul National University, College of Medicine, Seoul, Korea

[13]Department of Transdisciplinary Medicine, Seoul National University Hospital, Seoul, Korea

[14]Biomedical Research Institute, Seoul National University Hospital, Seoul, Korea

¶ These authors contributed equally to this work.

*Corresponding authors:

Seung-Hee Yang

Professor

Biomedical Research Institute, Seoul National University Hospital

Kidney Research Institute, Seoul National University College of Medicine

101 Daehak-ro, Jongno-gu, Seoul 03080, Korea

Tel: +82-2-2072-1724

E-mail: ysh5794@snu.ac.kr

Dohyun Han

Associate Professor

Department of Transdisciplinary Medicine, Seoul National University Hospital

101 Daehak-ro, Jongno-gu, Seoul 03080, Korea

Tel: +82-2-2072-4297

E-mail: hdh03@snu.ac.kr

Supplementary Fig. 1. Evaluation of the appropriateness of the 5/6 nephrectomy rat model.

(a) Changes in clinical factors, including body weight, blood pressure, blood urea nitrogen, blood creatinine, and urine protein/creatinine ratio were analyzed after 4- and 8-weeks of 5/6 nephrectomy operation. (b) Immunohistochemistry of kidney fibrosis (Periodic acid-Schiff [PAS], Masson's trichrome, Sirius-red), mitochondrial injury (superoxide dismutase-1 and cytochrome C), and acute kidney injury (neutrophil gelatinase-associated lipocalin) markers. (c) Quantification of positive areas of kidney fibrosis, mitochondrial injury, and acute kidney injury markers in the total area (n = 6). *$p < 0.05$; **$p < 0.005$; and ***$p < 0.001$.

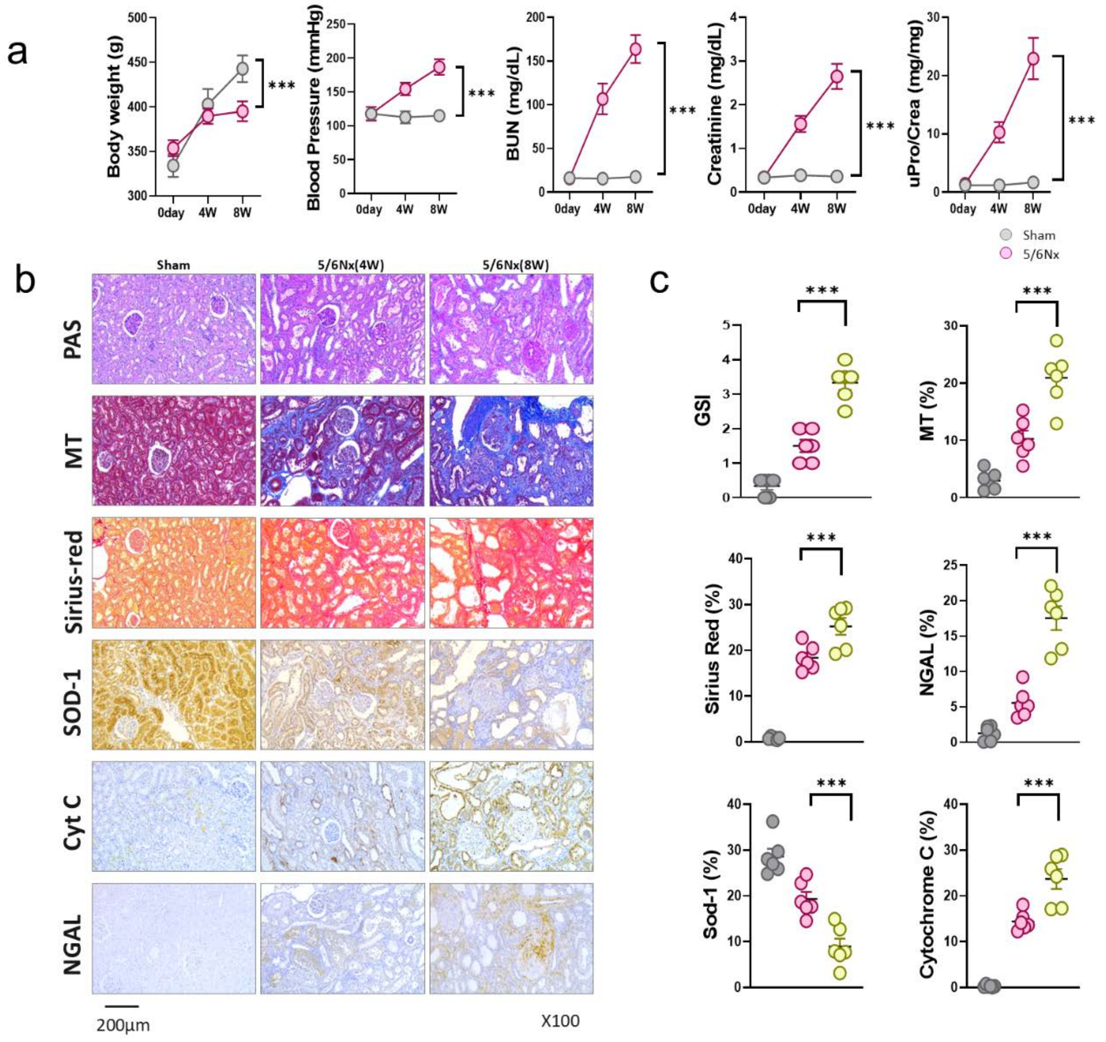

Supplementary Fig. 2. Relative abundance of kidney injury-associated proteins in hTECs

Fold change in protein expression in rTGF-β-treated hTECs in isobaric labeled proteomic data. The proteins are indicated in Figure 3B.

hTECs, human primary tubular epithelial cells; rTGF-β, recombinant transforming growth factor β

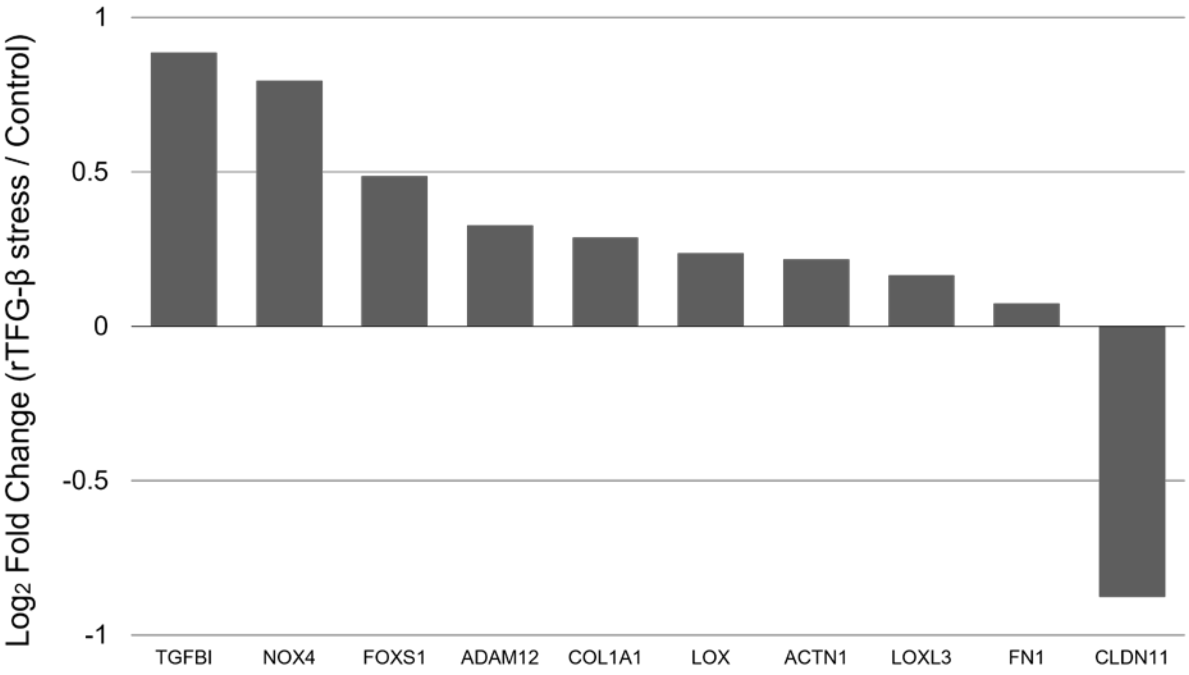

Supplementary Fig. 3. Protein abundance of kidney fibrosis-associated proteins in 5/6 Nx rat model.

Relative expression levels of proteins related to renal impairment and significantly expressed in the 5/6 Nx rat model. The proteins are shown in Figure 4B.

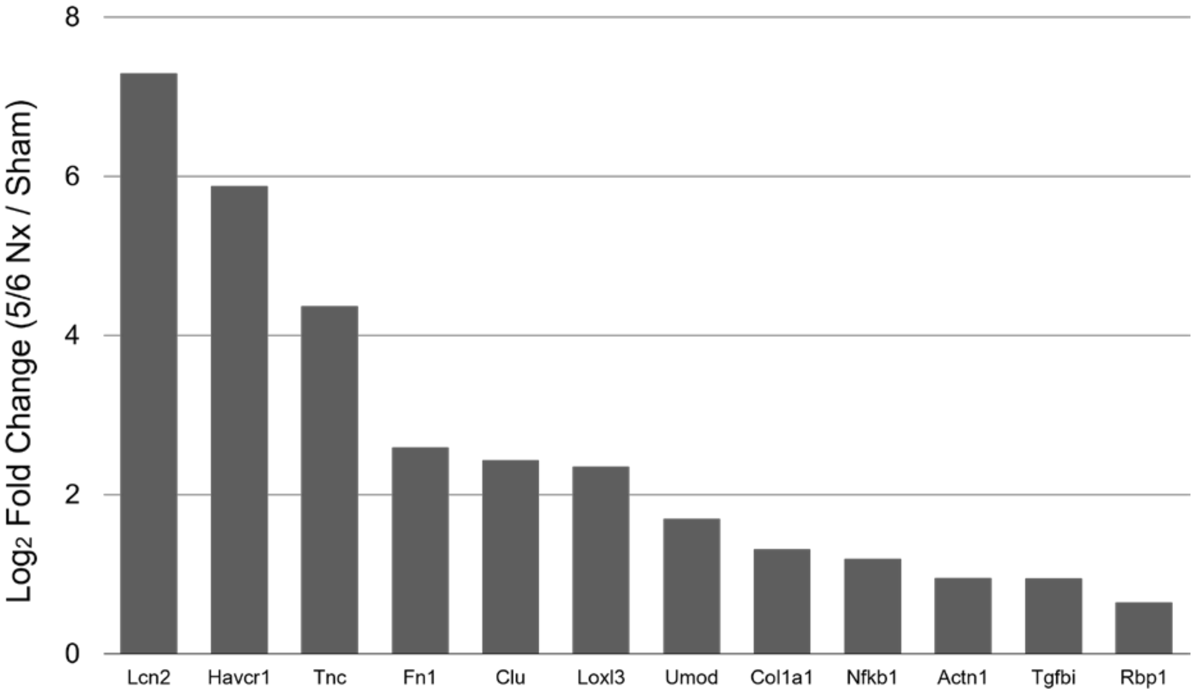

Supplementary Fig. 4. Relative expression of selected proteins from network analysis

The abundance of five selected proteins from TMT-labeled or label-free quantified proteomic data from hTECs and 5/6 Nx rats.

hTECs, human primary tubular epithelial cells; TMT, tandem mass tag

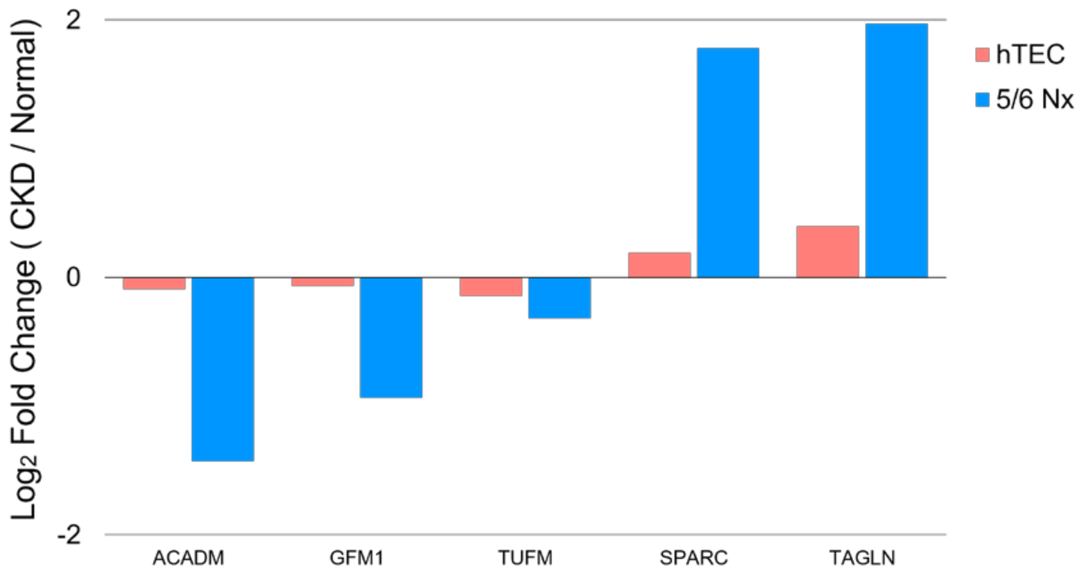

Supplementary Fig. 5. Correlation analysis between kidney tissue TAGLN expression and conventional laboratory variables.

The correlation between kidney tissue TAGLN expression and several conventional serum and urine (UPCR) laboratory variables was tested by Pearson's correlation analysis.

eGFR, estimated glomerular filtration rate; BUN, blood urea nitrogen; UPCR, urine protein-to-creatinine ratio; SBP, systolic blood pressure; DBP, diastolic blood pressure;

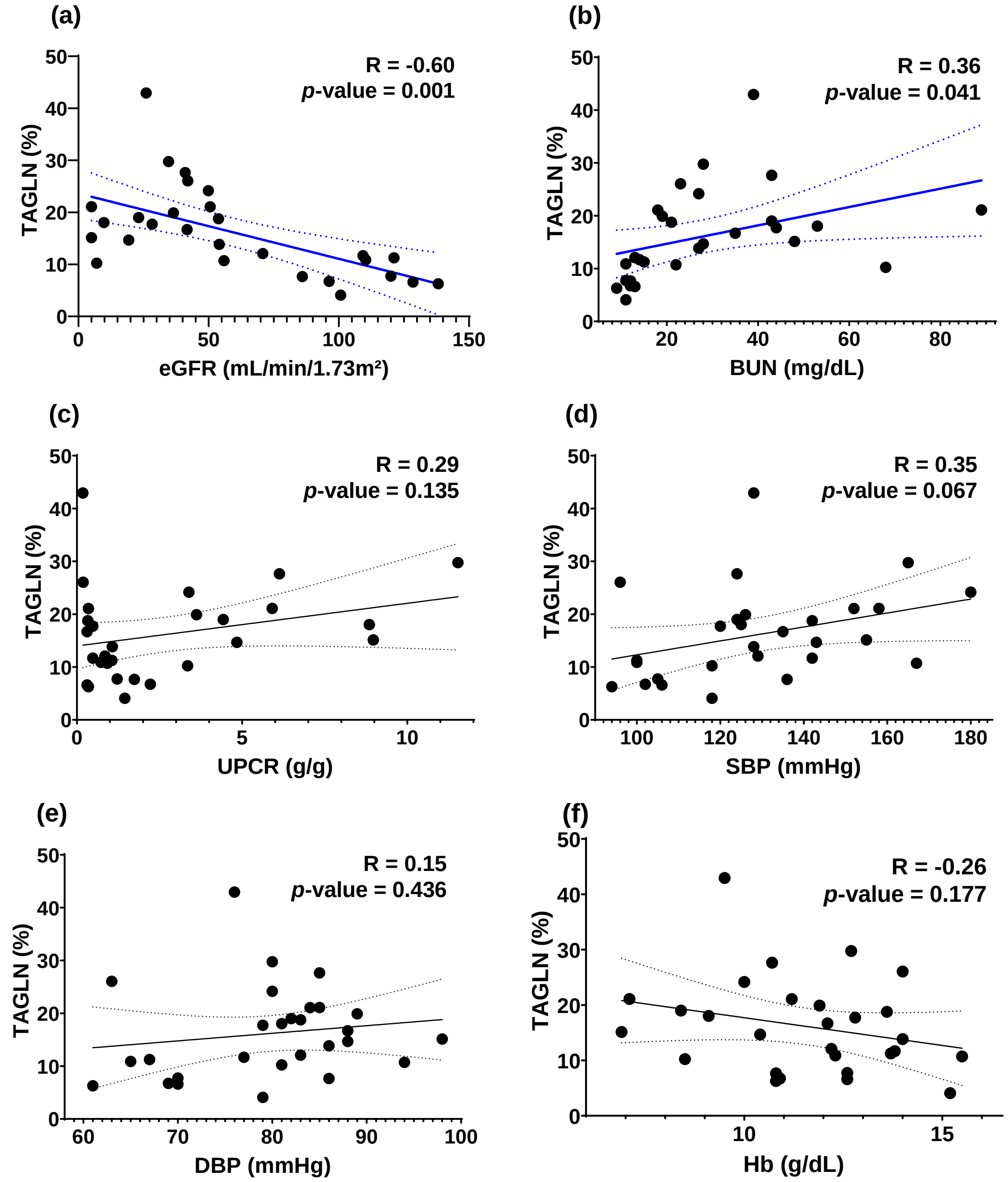

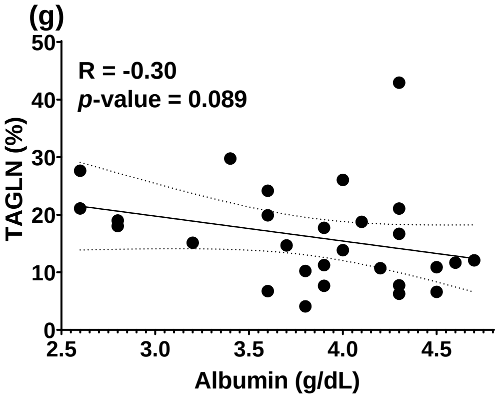
(g)
R = -0.30
p-value = 0.089
TAGLN (%)
Albumin (g/dL)
0
10
20
30
40
50
2.5
3.0
3.5
4.0
4.5

Supplementary Fig. 6. H_2O_2 induced reactive oxygen species stress was reduced by iTAGLN treatment.

ROS assay after 1 h H_2O_2 stress with or without iTAGLN treatment in hTECs (n=8) was performed. ROS, reactive oxygen species; iTAGLN, TAGLN-blocking peptide

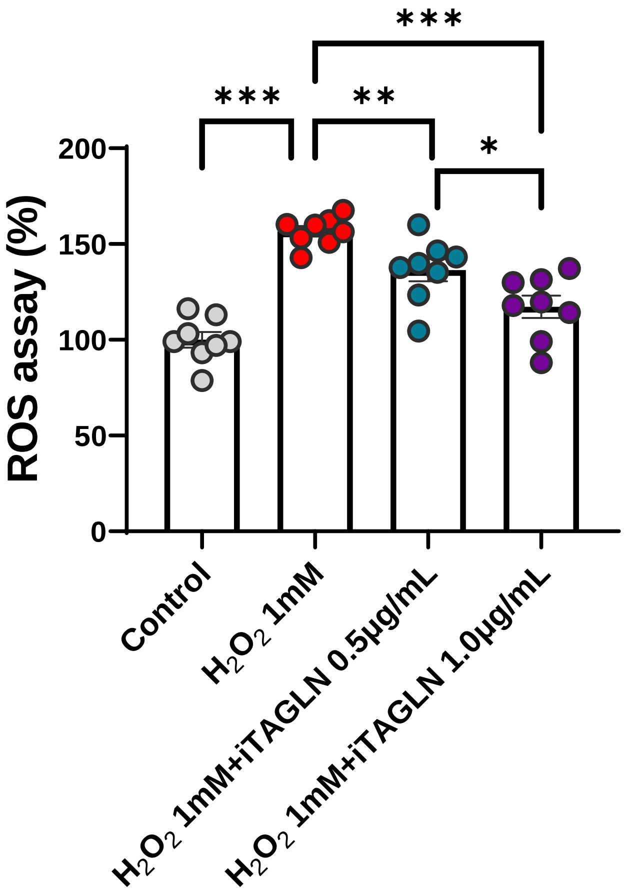

Supplement: Supplementary file 1 — Supplementary Information [file 12276_2024_1319_MOESM1_ESM.pdf]
